# Assessing Scaffold Diversity of Kinase Inhibitors Using Alternative Scaffold Concepts and Estimating the Scaffold Hopping Potential for Different Kinases

**DOI:** 10.3390/molecules22050730

**Published:** 2017-05-03

**Authors:** Dilyana Dimova, Jürgen Bajorath

**Affiliations:** Department of Life Science Informatics, B-IT, LIMES Program Unit Chemical Biology and Medicinal Chemistry, Rheinische Friedrich-Wilhelms-Universität, Dahlmannstr. 2, Bonn D-53113, Germany; dimova@bit.uni-bonn.de

**Keywords:** kinase inhibitors, analog series, compound-based scaffolds, analog series-based scaffolds, structural diversity, promiscuity, scaffold hopping

## Abstract

Publicly available kinase inhibitors provide a large source of information for structure–activity relationship analysis and kinase drug design. In this study, publicly available inhibitors of the human kinome were collected and analog series formed by kinase inhibitors systematically identified. Then, alternative scaffold concepts were applied to assess diversity and promiscuity of kinase inhibitors. Over the past two years, the number of publicly available kinase inhibitors with high-confidence activity data more than doubled, but coverage of the human kinome only slightly increased. Approximately 70% of current kinase inhibitors belonged to analog series. However, the detectable degree of promiscuity among these kinase inhibitors remained low. Approximately 76% of all inhibitors were only annotated with a single kinase, compared to ~70% two years ago. For many kinases, the assessment of scaffold diversity among their inhibitors and the distribution of differently defined scaffolds over analog series made it possible to assess scaffold hopping potential. Our analysis revealed that the consideration of conventional compound-based scaffolds most likely leads to an overestimation of scaffold hopping frequency, at least for compounds forming analog series.

## 1. Introduction

In medicinal chemistry, structural diversity of compound collections is frequently assessed at the level of core structures or scaffolds [1]. These terms are synonymously used, but the term scaffold often refers to a formalized and computer-accessible definition of core structures [1]. In medicinal chemistry, core structures are assessed in different ways, often subjectively, usually taking reaction information into account. Scaffolds are defined more formally for the systematic and consistent extraction of core structures, which generally depends on computational analysis [1]. Following a classical definition, scaffolds are obtained from compounds by removal of R-groups while retaining all ring systems and linker fragments between rings [2]. The resulting so-called Bemis–Murcko (BM) scaffolds have provided the basis for systematic scaffold comparisons [1,2]. To date, this scaffold definition is most widely applied in computer-aided medicinal chemistry [1]. Following this definition, each ring-containing compound yields a BM scaffold, which can represent varying numbers of compounds (containing the same scaffold). Thus, enumeration of BM scaffolds in data sets and calculation of compound-to-scaffold ratios is often used to assess structural diversity, which is expected to increase with increasing numbers of scaffolds and decreasing compound-to-scaffold ratios [1].

Going beyond the assessment of structural diversity, the scaffold concept is also of high relevance for the identification of novel active compounds. The term scaffold hopping refers to the computational search for active compounds containing different core structures [3,4]. The presence of different BM scaffolds in known active compounds used as search templates and newly identified hits is usually considered as an indicator of chemical novelty [4]. For computational screening methods, demonstrating BM scaffold hopping potential in benchmark calculations is generally regarded as a requirement for methodological ‘validation’, although the performance in benchmark studies rarely scales with the ability of computational screening to identify novel active compounds in prospective applications [4]. Importantly, different scaffolds might form a wide spectrum of structural relationships, from very closely to distantly related or even unrelated scaffolds. Thus, it is difficult to generalize scaffold hopping potential without paying close attention to relationships between scaffolds. Simply put, a scaffold hop is not like any other.

An important condition for successful scaffold hopping is the ability of target proteins to interact with different compound classes [5]. In computational scaffold hopping exercises, this is typically not considered. For the majority of pharmaceutically relevant targets, active compounds are available that contain large numbers of different BM scaffolds [5,6]. In these cases, active compounds represent many scaffold hops and newly identified hits may likely contain additional scaffolds. Thus, if more than just a few active compounds are available for a given target, evaluating their scaffold diversity makes it possible to estimate the scaffold hopping potential for this target [4]. 

In addition to structural analysis and scaffold hopping, the scaffold concept is also employed to map biological activities of compound series [1]. Therefore, target annotations of compounds sharing the same scaffold are assigned to the scaffold, which then represents the biological activity profile of the series at a higher level of structural abstraction. Although compounds are active—and typically not a formally defined scaffold—the organization of compound activities at the level of scaffolds is helpful for global structure–activity relationship (SAR) analysis. One can distinguish ‘active’ from ‘inactive’ scaffolds and scaffolds representing compounds that are active against the same target (single-target activity) from those that are associated with multi-target activities, i.e., promiscuous scaffolds [7].

In our current study, we focus on inhibitors of protein kinases, which play a prominent role among current pharmaceutical targets [8]. Over the past two decades, substantial efforts have been made to develop kinase inhibitors for therapy [8,9] and the search for novel inhibitors with new modes of action continues [9]. The human kinome comprises 518 kinases [10] and inhibitors for more than half of them have been reported [11]. In a systematic survey of publicly available inhibitors of the human kinome published two years ago, nearly 19,000 small molecule kinase inhibitors with available high-confidence activity data were identified that were directed against 266 human kinases [11]. These kinase inhibitors contained more than 7800 unique BM scaffolds [11,12]. Thus, there was a high degree of scaffold diversity among kinase inhibitors, but the majority of these scaffolds were involved in structural relationships [12]. Most kinase inhibitors were classical type I ATP site-directed compounds [13] while inhibitors with other modes of actions were rare [11].

For ATP site-directed inhibitors, promiscuity across different kinases is a heavily investigated issue [1,11] with high relevance for therapy [1,9]. However, on the basis of high-confidence activity data, ~70% of public domain inhibitors were only annotated with a single kinase and only ~1% of the inhibitors were known to be active against five or more kinases [11]. Similarly, more than 70% of BM scaffolds from kinase inhibitors were single-target scaffolds and only ~2% were highly promiscuous scaffolds [12].

BM scaffolds have dominated computational scaffold analysis for more than two decades [1]. Recently, we have introduced a different scaffold concept for computational analysis to further increase the utility of scaffolds for medicinal chemistry applications [14]. As indicated by their name, analog series-based (ASB) scaffolds were designed to represent compound series and take reaction information into account [14]. This represents a major difference compared to BM scaffolds that originate from individual compounds and are defined following a structural hierarchy. In a systematic survey, ASB scaffolds were isolated from 15,625 different series of active compounds (yielding one ASB scaffold per series). These series produced 22,224 BM scaffolds [15]. With very few exceptions, ASB and BM scaffolds originating from the same analog series were structurally distinct, but about a third of the ASB scaffolds contained a BM scaffold as a substructure [15].

Herein, we revisit the analysis of kinase inhibitors employing the ASB scaffold concept. For publicly available compounds active against the human kinome, scaffold diversity and promiscuity was assessed on a per-kinase basis. By comparing the diversity of ASB and BM scaffolds, the scaffold hopping potential for different kinases was evaluated on the basis of alternative scaffolds concepts.

## 2. Results and Discussion

### 2.1. Alternative Scaffold Concepts

**Figure 1:**
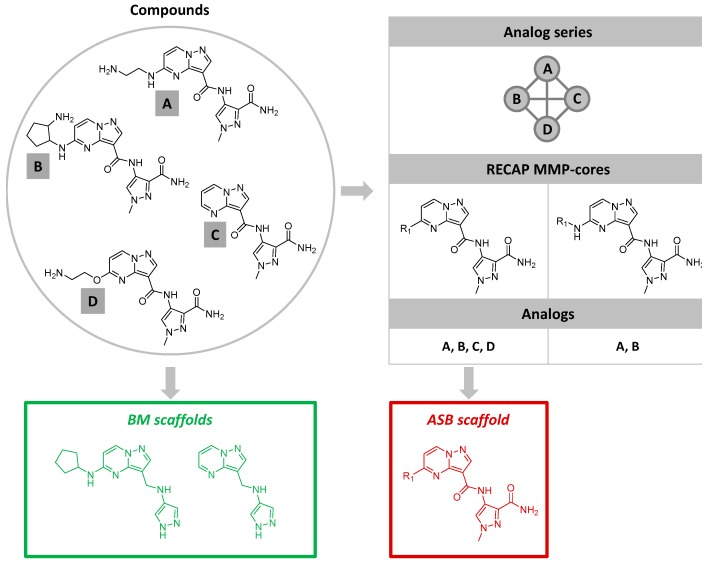
The generation of BM and ASB scaffolds is illustrated. On the left, the structures of four exemplary compounds (**A**–**D**) forming an analog series are shown. On the right, a network representing the MMP relationships within the series is displayed. In addition, two RECAP-MMP cores originating from the series are depicted. The core that is shared by all four analogs represents the ASB scaffold (red). Furthermore, BM scaffolds extracted from analogs (**A**–**D**) are shown (green).

The generation of BM and ASB scaffolds is illustrated in Figure 1. BM scaffolds are obtained following a molecular hierarchy. Substituents with exocyclic bonds are removed from compounds while ring systems and aliphatic linkers between rings are retained. Thus, from each ring-containing compound, a BM scaffold is extracted.

By contrast, ASB scaffolds are derived from analog series. These series are systematically identified by applying the matched molecular pair (MMP) formalism [16]. An MMP is defined as a pair of compounds that are only distinguished by a chemical modification at a single site [16]. Thus, each MMP contains a conserved MMP core and a pair of exchanged substituents. For ASB scaffold analysis, MMPs are generated on the basis of retrosynthetic (RECAP) rules [17] to systematically fragment bonds. From the resulting RECAP-MMPs [18] of a set of active compounds, a network is constructed in which nodes represent compounds and edges pairwise RECAP-MMP relationships [19]. In this network, each separate (disjoint) cluster represents a unique series of analogs [13], which can be easily extracted. From these analog series, ASB scaffolds are isolated. A series often contains multiple RECAP-MMP cores and a search is carried out for a core that covers all RECAP-MMP relationships within a series. If such a core exists, it represents the ASB scaffold of the series [14]. Thus, depending on the MMP relationships within a given series, an ASB scaffold may or may not be obtained. If more than one qualifying RECAP-MMP core is present, the largest one is selected. An ASB scaffold consists of all conserved structural elements of an analog series and hence captures series-specific structural information. Furthermore, the ASB scaffold contains a single substitution site that differentiates analogs comprising a series. All of these analogs, as well as new ones, can be generated from the ASB scaffolds following applicable RECAP rules.

### 2.2. Inhibitor and BM Scaffold Statistics

A total of 43,331 kinase inhibitors with available high-confidence data were identified in the current release of ChEMBL [20]. These inhibitors were active against 286 human kinases from 12 different groups. Previously, in 2015, 18,653 inhibitors with high-confidence activity data were available that were active against 266 kinases [11]. Thus, public domain kinase inhibitors have more than doubled over the past two years, but coverage of the human kinome has only slightly increased, with 20 new kinase targets. The number of distinct BM scaffolds contained in kinase inhibitors also more than doubled over the past two years, with 16,516 compared to 7823. However, the compound-to-scaffold ratio remained almost constant, with on average 2.6 compounds per scaffold. Thus, most of the newly reported inhibitors also contained new BM scaffolds, indicating increasing structural diversity at the level of these scaffolds.

### 2.3. Analog Series and ASB Scaffolds

Next analog series formed by currently available kinase inhibitors were systematically determined. We detected 4172 unique series containing 30,176 inhibitors (~70% of all) with on average seven analogs per series that were active against 261 human kinases. Thus, despite a high degree of BM scaffold diversity, 70% of the inhibitors were part of analog series. The 4172 series yielded 11,054 different BM scaffolds with on average 2.6 scaffolds per series. Furthermore, ASB scaffolds were derived from 2836 series (68% of all). These series contained 4492 BM scaffolds. The ASB scaffolds (one and only one for each qualifying series) covered 9643 inhibitors that were active against 231 kinases. On average, an ASB scaffold represented 3.4 compounds.

Analog series yielded ASB scaffolds if a RECAP-MMP core existed that represented all structural relationships within a series and, in addition, if all structural modifications were confined to a single substitution site in this core. These requirements rationalized the numerical difference between analog series and resulting ASB scaffolds. Since ASB scaffolds represented compound series, their compound-to-scaffold ratio was usually higher than for BM scaffolds, as further discussed below.

### 2.4. Compound and Scaffold Promiscuity

Although the number of available kinase inhibitors more than doubled over the past two years, the proportion of inhibitors that were only annotated with a single kinase further increased to 76.5% compared to 70% two years ago. Thus, on the basis of high-confidence activity data, only 23.5% of all currently available human kinase inhibitors were promiscuous. Moreover, only 504 inhibitors (~1%) were annotated with five or more kinases. Similar trends were observed for kinase inhibitors that produced ASB and BM scaffolds. In total, 23.5% of these inhibitors were promiscuous and were distributed across 829 ASB and 1130 BM scaffolds. These scaffolds represented on average 2.7 (ASB) and 2.0 (BM) promiscuous kinase inhibitors. 

**Figure 2:**
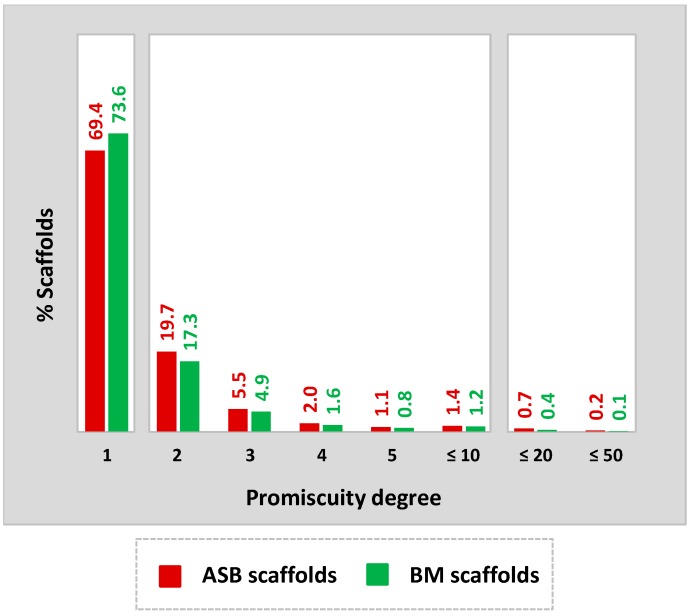
The distribution of 2836 kinase inhibitor ASB (red) and 4492 corresponding BM scaffolds (green) over increasing promiscuity degrees is reported.

**Figure 3:**
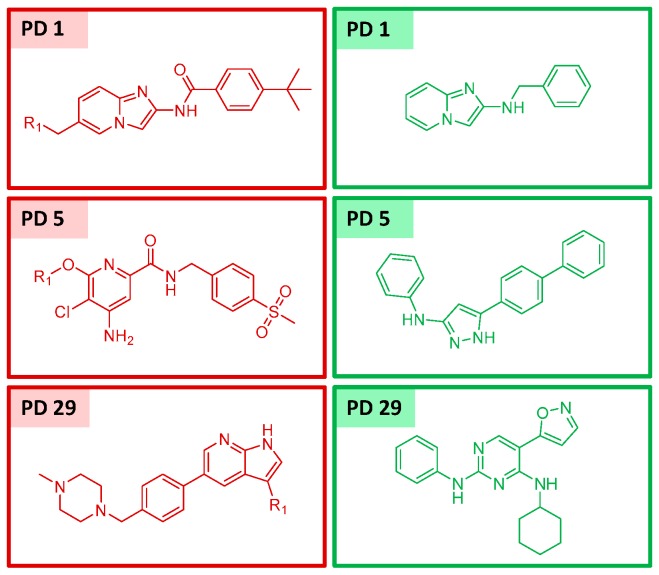
Shown are exemplary ASB (red) and BM scaffolds (green) with increasing promiscuity degree (PD).

Promiscuity was generally assessed at the level of BM and ASB scaffolds after assigning all unique kinase annotations of inhibitors represented by a given scaffold to this scaffold. Figure 2 reports the distribution of ASB and corresponding BM scaffolds (originating from the same series) over increasing promiscuity degrees (i.e., total number of kinase annotations). It was found that 73.6% of BM and 69.4% of ASB scaffolds were single-target scaffolds. By contrast, 0.8% of BM and 1.1% of ASB scaffolds were annotated with 5 targets and 1.2% and 1.4% with 6–10. Thus, the degree of scaffold promiscuity was overall low for BM and ASB scaffolds, but slightly higher for ASB scaffolds, as expected for series-based scaffolds. However, the overall low degree of promiscuity among ASB scaffolds, with 69.4% single-target scaffolds, also indicated that the majority of analog series were only annotated with an individual kinase. However, individual ASB and BM scaffolds with large promiscuity degrees were also detected, as shown in Figure 3. 

**Figure 4:**
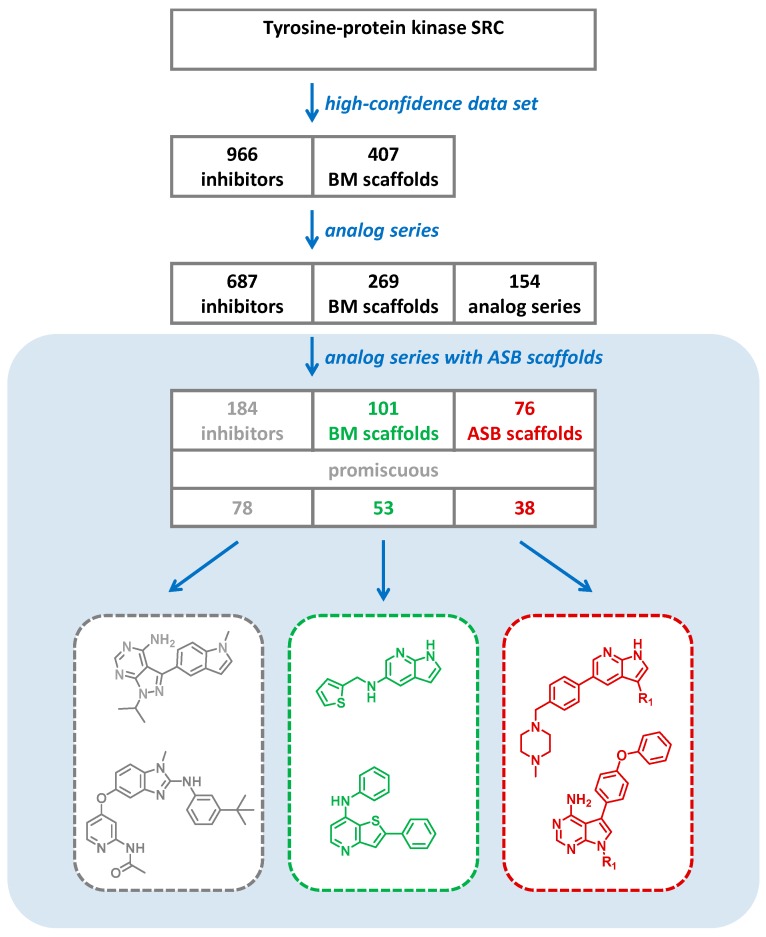
For the Src kinase, the distribution of inhibitors and scaffolds is provided. In addition, the number of promiscuous inhibitors and scaffolds isolated from analog series producing an ASB scaffold is reported. Exemplary compounds (gray), ASB (red), and BM scaffolds (green) are shown.

As a representative example for a popular kinase target, inhibitors and scaffolds are reported for tyrosine kinase Src. The distribution of inhibitors and scaffolds with high-confidence activity data, analog series, and analog series yielding ASB scaffolds is shown in Figure 4. In total, there were 184 Src inhibitors producing 101 BM and 76 ASB scaffolds. In addition, there were 78 promiscuous kinase inhibitors yielding 53 and 38 promiscuous BM and ASB scaffolds, respectively. 

### 2.5. Scaffold Distribution

The 2836 ASB scaffolds represented well-defined analog series comprising nearly 10,000 kinase inhibitors. These analog series contained a total of 4492 BM scaffolds (see Section 2.3.). For this subset of inhibitors, ASB and BM scaffolds could be directly compared. For 54.9% of the series yielding an ASB scaffold, multiple BM scaffolds were detected. More than 40% of these analog series produced two or three BM scaffolds, and individual series were found to contain 10 to 20 BM scaffolds. Thus, analog series producing an ASB scaffold yielded varying numbers of BM scaffolds. Furthermore, more than half (63.2%) of all 4172 analog series of kinase inhibitors we identified—including those without ASB scaffolds—produced multiple BM scaffolds. 

**Figure 5:**
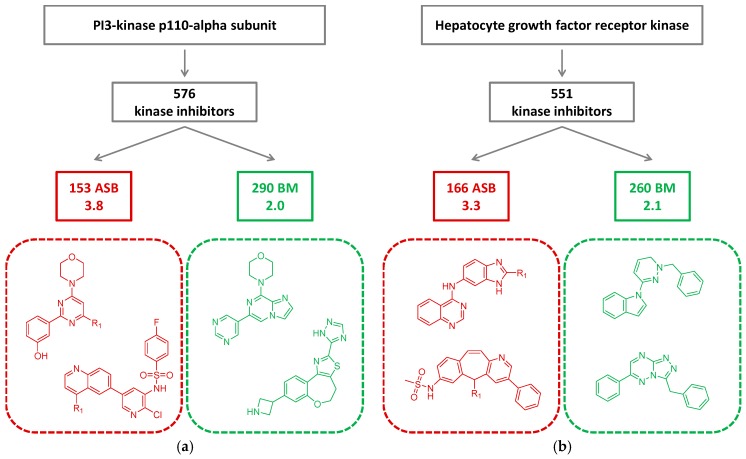
For two kinases, (**a**) PI3-kinase p110-alpha subunit and (**b**) hepatocyte growth factor receptor kinase, the number of inhibitors yielding ASB scaffolds, the number of ASB scaffolds, and the number of BM scaffolds contained in these inhibitors are reported. In addition, compound-to-scaffold ratios are given for ASB and BM scaffolds. Exemplary ASB (red) and BMscaffolds (green) are shown.

In Figure 5, ASB and BM scaffolds are compared for inhibitors of two different kinases that formed analog series producing ASB scaffolds. In each case, the compound-to-scaffold ratio was higher for ASB than BM scaffolds. For the PI3-kinase p110-alpha subunit (Figure 5a), the ratio was 3.8 and 2.0 for ASB and BM scaffolds, respectively, and for hepatocyte growth factor receptor kinase (Figure 5b), the ratio was 3.3 (ASB) and 2.1 (BM). 

### 2.6. Implications for Scaffold Hopping

**Figure 6:**
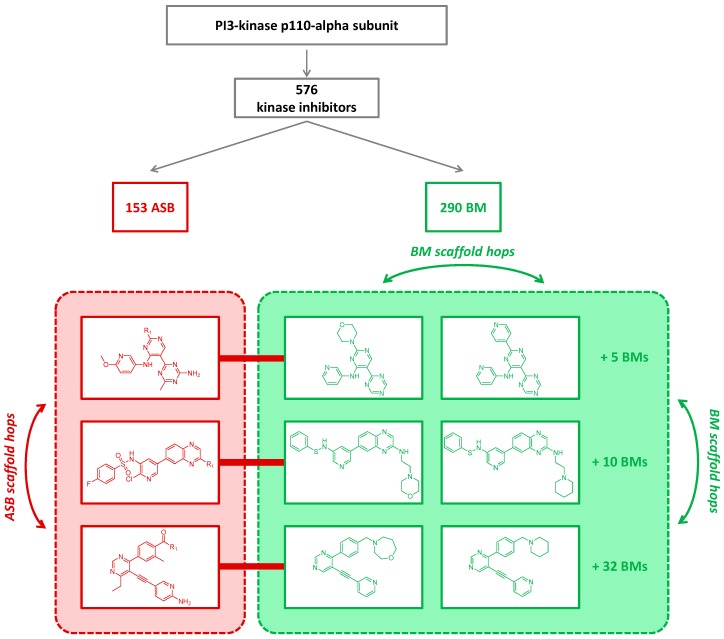
For inhibitors of the PI3-kinase p110-alpha subunit according to Figure 5a, three exemplary ASB scaffolds are shown (red). For each of the corresponding analog series, two exemplary BM scaffolds are provided (green). In each case, the number of additional BM scaffolds originating from the series is reported. Arrows indicate possible scaffold hops.

A series of structural analogs contains a shared core structure and should thus produce a single scaffold, as exemplified by the 1-to-1 correspondence of analog series and ASB scaffolds. However, this depends on the way scaffolds are defined. Accordingly, a series of analogs might produce multiple compound-based BM scaffolds, as reported herein. Such scaffolds are similar—and might themselves be regarded as analogs—but are considered distinct entities in scaffold hopping calculations [4]. Thus, if systematic scaffold hopping studies are carried out on kinase inhibitors, BM scaffold hops would be detectable within more than half of all series of analogs that are currently available. As illustrated in Figure 6, this would lead to a statistical overestimation of scaffold hopping potential for kinase targets for which inhibitor analog series are available. In the example in Figure 6, the three analog series-producing ASB scaffolds contain 7, 12, and 34 BM scaffolds, respectively. Thus, focusing only on these series, three ASB scaffold hops would be possible, but an abundance of BM scaffolds hops, which would bias the statistics of scaffold hopping analysis. Clearly, detecting scaffold hopping events for kinase inhibitors within analog series on the basis of multiple BM scaffolds would not be very meaningful from a chemical perspective.

Considering scaffold hopping on a large scale, the use of ASB scaffolds would currently only cover half of the available series of kinase inhibitors, but would provide a more realistic assessment of scaffold hopping potential for the corresponding subset of kinases. For analog series, scaffold hopping potential can be estimated and differentiated based on these considerations. However, currently 13,155 kinase inhibitors (30.3% of all) are available that do not have structural analogs; a perhaps unexpectedly large proportion from a medicinal chemistry perspective. Structural relationships between such (singleton) compounds and their scaffolds that might be detected in scaffold hopping calculations can only be assessed on a case-by-case basis, for example, with the aid of compound-based BM scaffolds or on the basis of maximum common substructures that are also suitable for pairwise comparisons.

### 2.7. Implications for Drug Discovery

The number of available kinase inhibitors continues to grow rapidly and further increases the knowledge base for kinase drug discovery. On the basis of high-confidence activity data, about 75% of all inhibitors are only annotated with a single kinase, despite the substantial increase in new inhibitors. Gradually lowering data confidence criteria likely increases promiscuity degrees of inhibitors, but for global analysis, we strictly adhere to highest confidence levels. The predominance of single-target inhibitors observed under these conditions implies that many newly published inhibitors might have been tested only against a limited number of kinases other than their primarily intended target in high-confidence assays, leaving much room for follow-up investigations. Inhibitor growth is also accompanied by further increases in BM scaffold diversity. However, as demonstrated by comparison of BM and ASB scaffolds, care should be taken not to overestimate structural diversity among kinase inhibitors on the basis of BM scaffold diversity. As discussed above, this also applies to computational scaffold hopping exercises.

## 3. Materials and Methods

Publicly available inhibitors covering the human kinome were extracted from ChEMBL [20] release 22. Inhibitors with reported direct interactions (target relationship type “D”) with human kinase targets at the highest confidence level (target confidence score 9) were extracted. Two different types of potency measurements were considered, including equilibrium constants (K_i_) and IC_50_ values. Approximate measurements such as “>”, “<”, or “~” were not considered. For compounds with multiple K_i_ or IC_50_ measurements against the same target, the geometric mean of all potency values was calculated as the final potency annotation, provided these values fell within the same order of magnitude. Although IC_50_ and K_i_ values cannot be directly compared, for the purpose of our analysis, focusing on target annotations, it was possible to combine these high-confidence measurements for kinase inhibitors. Kinases were designated and assigned to groups following the UniProt classification scheme [21]. For promiscuity analysis of inhibitors, only kinase targets were considered. Analog series and BM or ASB scaffolds were generated using previously published in-house developed (analog series, ASB scaffolds) or adapted (BM scaffolds) methods [2,14,19], as explained in the text.
